# Generalized Ketogenic Diet Induced Liver Impairment and Reduced Probiotics Abundance of Gut Microbiota in Rat

**DOI:** 10.3390/biology13110899

**Published:** 2024-11-04

**Authors:** Ge Song, Dan Song, Yongwei Wang, Li Wang, Weiwei Wang

**Affiliations:** Key Laboratory of Grain and Oil Biotechnology, Academy of National Food and Strategic Reserves Administration, Beijing 100037, China; sg@ags.ac.cn (G.S.); sd@ags.ac.cn (D.S.); wyw@ags.ac.cn (Y.W.)

**Keywords:** generalized ketogenic diet, lipoidosis, gut microbiota, apparent metabolizable energy

## Abstract

The ketogenic diet is becoming an assisted treatment to control weight, obesity, and even type 2 diabetes. However, there has been no scientific proof supporting that the ketogenic diet is absolutely safe and sustainable. In this study, Sprague–Dawley (SD) rats were fed different ratios of fat to carbohydrates under the same apparent metabolizable energy level to evaluate the effects of a ketogenic diet on healthy subjects. The results showed that the ketogenic diet could relatively sustain body weight and enhance the levels of serum alanine aminotransferase (ALT) and serum alkaline phosphatase (SAP), leading to more moderate lipoidosis and milder local non-specific inflammation in the liver compared with the high-carbohydrate diet. In addition, the abundance of probiotic strains such as *Lactobacillus*, *Lactococcus*, and *Faecalitalea* were reduced with the ketogenic diet in rats, while an abundance of pathogenic strains such as *Anaerotruncus*, *Enterococcus*, *Rothia*, and *Enterorhabdus* were increased with both the ketogenic diet and the high-carbohydrate diet. This study suggests that the ketogenic diet can lead to impairments of liver function and changed composition of the gut microbiota in rats, which to some extent indicates the danger of consuming a generalized ketogenic diet.

## Simple Summary

The generalized ketogenic diet is increasingly popular for weight loss and managing conditions like obesity and type 2 diabetes. However, concerns about its long-term safety remain. Our study aimed to investigate the effects of this diet on healthy rats, comparing it to a high-carbohydrate diet. We found that while the generalized ketogenic diet helped maintain body weight, it also raised liver enzyme levels, suggesting possible liver damage. Additionally, it altered gut bacteria, reducing beneficial microbes and increasing potentially harmful ones. These findings indicate that the generalized ketogenic diet may not be entirely safe and could have negative health implications. Understanding these effects is crucial for guiding public health recommendations and dietary advice.

## 1. Introduction

The ketogenic diet (KD) is a special dietary pattern which, in a broad sense, is a low-carbohydrate, adequate-protein, and high-fat diet; the macronutrient ratio of a strict ketogenic diet is fat:protein:carbohydrates = 7:2:1 [1]. In the 1920s, this diet was primarily used to treat epilepsy in children [2,3]. This therapeutic dietary pattern provides sufficient protein and calories through the consumption of high-fat food and the exclusion of starchy cereals and fruits [4]. In the circumstances of lower carbohydrate intake, the liver converts fat into ketones and fatty acids as replacements for glucose, which has recently been generally considered as a novel way to control body weight and blood glucose levels [5,6].

Several studies have investigated the physiological effects of the KD on both healthy subjects [7] and those with illnesses, including those suffering from obesity [8], type 2 diabetes [9], polycystic ovary syndrome [10], and even cancer [11,12]. As stated above, the KD was considered effective in weight and blood glucose control in the diseased population. For the healthy population, the mass of subcutaneous fat was decreased. However, no change was observed in the fat distribution of healthy people in 6 weeks of research. Furthermore, the benefits of combining the KD and other treatments were also studied. Combining the KD and melatonin could inhibit vincristine and cisplatin drug resistance in a breast cancer animal model [13]. Up to now, the KD has been widespread as an intervention treatment for body weight control and diabetes, especially in Western countries. However, despite promoting rapid weight loss and improving certain health biomarkers, it raises concerns due to its potential to increase LDL cholesterol levels and possible long-term health consequences, leading to an ongoing debate between supporters and critics regarding its effectiveness, sustainability, and safety compared to plant-based diets [14]. Moreover, for sub-healthy people, it is very difficult to implement a strict ketogenic diet, and also, there have been similar cases evaluating the ketogenic diet in animal experiments. Therefore, we reduced the percentage of fat in the strictly ketogenic diet and created a “generalized ketogenic diet” to investigate the effects of a high-fat diet on lipidemia, liver histopathology, colon length, and gut microbiota in rats.

## 2. Materials and Methods

### 2.1. Ethics Statement

The study was launched strictly according to the Animal Ethics Committee Guidelines (registration number: 2018R02) of the Academy of National Food and Strategic Reserves Administration and conformed to the ARRIVE Guidelines for Reporting Animal Research.

### 2.2. Animals and Diets

Male Sprague–Dawley (SD) rats (303.59 ± 0.30) were purchased (Beijing HFK Bioscience Co., Ltd., Beijing, China) at 10 weeks of age. They were housed in individual ventilated cages (2 rats per cage) at a temperature of 23 ± 2 °C and 50% relative humidity with a 12 h light−dark cycle (SPF condition). After 1 week of acclimation, 50 SD rats were randomly divided (*n* = 10) and were given free access to one of the five isoenergetic (apparent metabolizable energy, AME) diets based on body weight for 8 weeks. The diets of the five groups were formulated with different fat/carbohydrate (F/C) ratios, and brown rice was the sole source of carbohydrates: (1) F10: 10% energy from fat, 70% energy from carbohydrates; (2) F20: 20% energy from fat, 60% energy from carbohydrates; (3) F30: 30% energy from fat, 50% energy from carbohydrates; (4) F40: 40% energy from fat, 40% energy from carbohydrates; (5) F50: 50% energy from fat, 30% energy from carbohydrates (generalized ketogenic diet). All diets (formulas are demonstrated in Table 1) were produced by TROPHIC (TROPHIC, Nantong, China).

### 2.3. Metabolizable Energy Determination

The apparent metabolizable energy (AME) levels of six main ingredients in the experimental diets, including brown rice, casein, soybean oil, sucrose, dextrin, and celluarose, were determined. Thirty-five male, 8-week-old SD rats were randomly divided into 7 groups (*n =* 5). The rats were fed in metabolic cages for 1 week of acclimation and 16 days of experimental treatment. The feces of the rats was collected during the last 6 days. Rats in the control group were fed the AIN-93M basal diet, and rats in each treatment group were fed a diet comprising 30% ingredient sample and 70% basal diet. A set of algorithms was used to measure the digestibility and metabolism energy of each ingredient sample. In cohort studies, we usually need to maintain the total energy consistency between the treatment groups to conduct the study due to the difficulties of calculating effective energy in human samples. In this study, fecal energy and urine energy in rats were collected and calculated, so that the AME levels of the 5 diets (F10, F20, F30, F40, F50) were consistent for the study.

AME = IE − (FE + UE)(1)

IE stands for in total energy; FE and UE stand for fecal energy and urinary energy, respectively [15].

### 2.4. Preparation of Tissue and Serum Sample

After food deprivation for 12 h, the rats were sacrificed by carbon dioxide inhalation. Serum was obtained by whole-blood centrifugation at 2000× *g* for 15 min at 4 °C. Kidneys and livers were obtained and weighted, and colon lengths were measured.

### 2.5. Analysis of Serum

Liver histopathological determination was analyzed according to the previous method [16]. Alanine aminotransferase (ALT), aspartic aminotransferase (AST), serum alkaline phosphatase (SAP), total protein (TP), blood urea nitrogen (BUN), serum total triglyceride (TG), total cholesterol (TC), low-density lipoprotein cholesterol (LDL), and high-density lipoprotein cholesterol (HDL) were measured by a biochemistry analyzer (Roche Modular, Roche, WA, USA).

### 2.6. 16S rRNA Sequencing

The total genome DNA of the samples was extracted by CTAB/SDS. DNA concentration and purity was monitored on 1% agarose gels. DNA samples were diluted to 1 ng/μL with sterile water. The V4 hypervariable region of 16S rRNA genes was amplified using the specific primer with the barcode. All PCR reactions were carried out in 30 μL reactions with 15 μL of Phusion^®^ High-Fidelity PCR Master Mix (New England Biolabs (NEB), Beijing, China), 0.2 μM of forward and reverse primers, and 10 ng template DNA. Thermal cycling consisted of initial denaturation at 98 °C for 1 min, 30 cycles of denaturation at 98 °C for 10 s, annealing at 50 °C for 30 s, elongation at 72 °C for 30 s, and finally, 72 °C for 5 min. We mixed the same volume of 1× loading buffer (contained SYB green) with PCR products and extracted electrophoresis on 2% agarose gel. Samples with bright main strips between 400 and 450 bp were chosen for further experiments. PCR products were mixed in equidensity ratios and purified with the GeneJET Gel Extraction Kit (Thermo Scientific, Beijing, China).

Sequencing libraries were generated by using the NEB Next^®^ Ultra™ DNA Library Prep Kit for Illumina (NEB, Beijing, China) with the manufacturer’s recommendations and indexcodes, and their quality was assessed by the Qubit@ 2.0 Fluorometer (Thermo Scientific) and Agilent Bioanalyzer 2100 system (Lexington, MA, USA), then sequenced on an Illumina HiSeq platform (San Diego, CA, USA).

### 2.7. Statistical Analysis

Values are displayed as means ± SEM. One-way ANOVA was applied to analyze data including growth performance, serum samples, and tissue weight/length using Statistical Product and Service Solutions (SPSS 23.0). Statistical significance among groups was determined by the Student–Newman–Keuls comparison test. Paired-end reads from the original DNA fragments were merged by FLASH 1.2.11. The QIIME software package (Quantitative Insights into Microbial Ecology 2.0) was applied for sequencing analysis, while in-house Perl scripts were applied to analyze alpha- (within samples) and beta- (among samples) diversity. Reads were filtered by QIIME quality filters for operational taxonomic units (OTUs). Sequences with ≥97% similarity were determined to be the same OTUs. The RDP classifier was used to annotate taxonomic information for each representative sequence. *p* ≤ 0.05 was considered as significant.

## 3. Results

### 3.1. Response of Growth Performance, Tissue Weight, and Colon Length Changes to Different Dietary Fat/Carbohydrate Ratios

**Figure 1:**
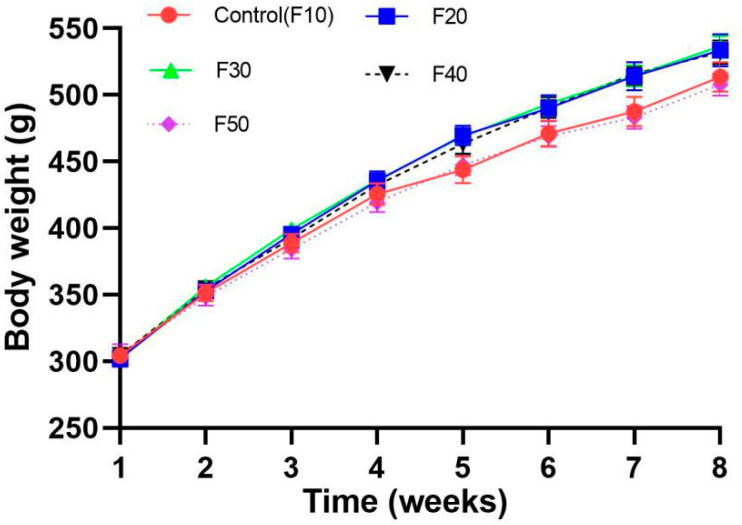
Body weight of SD rats (*n =* 10) fed diets with different ratios of fat/carbohydrates (10/70, 20/60, 30/50, 40/40, and 50/50).

The body weights (BWs) of the SD rats slightly increased during the experimental period (Figure 1). However, there were no significant differences in body weight gain among all the rats (*p* > 0.05). However, with the increasing F/C ratio in diets, the colon lengths of the rats were increased dose-dependently. Colon length was significantly higher in rats of the F40 (40/40) and F50 (50/50) groups compared with those in rats of the F20 (10/70) and F30 (20/60) groups, and remarkably higher than those in rats of the F10 (50/50) group (*p* < 0.001, Table 1).

### 3.2. Response of Liver Impairment to Different Dietary Fat/Carbohydrate Ratio

**Figure 2:**
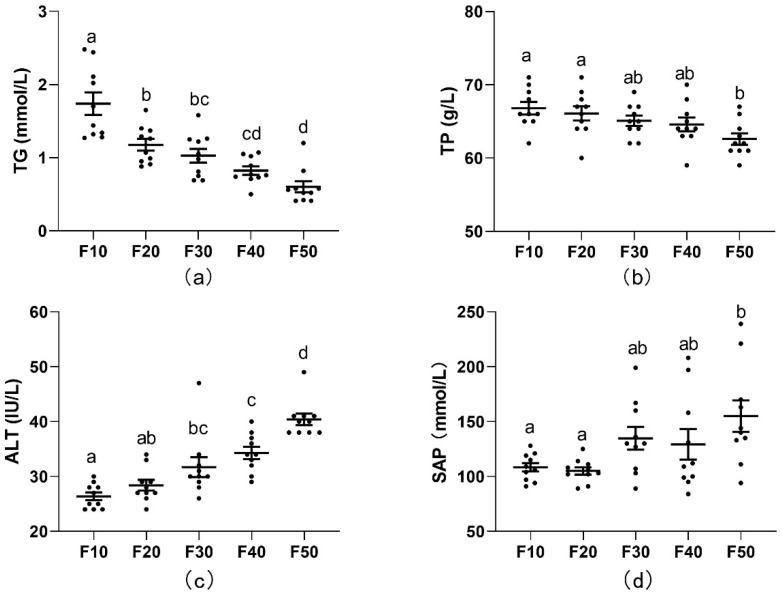
Serum total triglyceride (**a**), total protein (**b**), serum alanine aminotransferase (**c**), and serum alkaline phosphatase (**d**) levels of SD rats fed diets with different ratios of fat/carbohydrates (10/70, 20/60, 30/50, 40/40, and 50/50). Values are shown as means ± SEM, *n =* 10. Means in a list without a common letter differ, *p* < 0.05.

The serum total triglyceride (TG) level was decreased, and serum alanine aminotransferase (ALT) was increased dose-dependently (*p* < 0.05; Figure 2a,c) with the increasing ratio of F/C. Meanwhile, the serum total protein (TP) level was significantly lower and the serum alkaline phosphatase (SAP) content was significantly higher in rats of the F50 group compared with those in rats of the F10 group (*p* < 0.05; Figure 2b,d).

**Figure 3:**
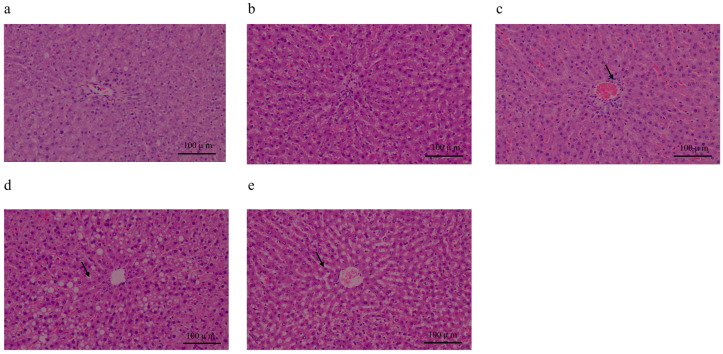
Light photomicrographs of live tissue of SD rats fed diets with different ratios of fat/carbohydrates (10/70, 20/60, 30/50, 40/40, and 50/50) for 8 weeks. (Hematoxylin–eosin-stained, captured in original magnification of 200×), *n =* 10. (**a**) 10/70 (F10). (**b**) 20/60 (F20). (**c**) 30/50 (F30). (**d**) 40/40 (F40). (**e**) 20/60 (F50). Arrows present pathological changes of rats.

No abnormality was observed in the livers of rats in groups F10 and F20 (Figure 3a,b). However, small numbers of infiltrating inflammatory cells (Figure 3c) were found in the livers of group F30 (30/50), while lipid droplets were found in the hepatocytes and 33% hepatic steatosis cells of all the cells in the field of vision (*n =* 10) in the livers of rats in the F40 (40/40) and F50 (50/50) groups (Figure 3d,e). Moderate lipoidosis and mild local non-specific inflammation were observed in the high-F/C-ratio group.

### 3.3. Response of Relative Abundance and Composition of Gut Flora to Different Dietary Fat/Carbohydrate Ratios

**Figure 4:**
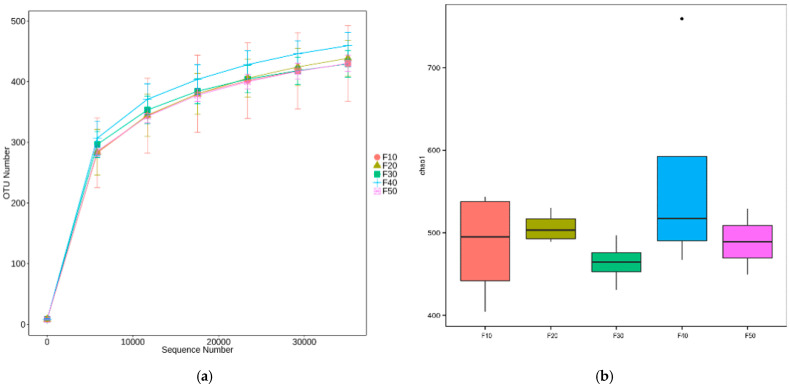
Alpha diversity of F10 (control) SD rats and SD rats fed diets with different ratios of fat/carbohydrates (10/70, 20/60, 30/50, 40/40, and 50/50), *n =* 4. (**a**) Rarefaction curve. (**b**) Chao1 index levels.

A rarefaction curve (Figure 4a) was achieved with a saturation plateau, which indicated that most of the OTUs in the samples were captured. The Chao1 index (Figure 4b) indicated that there were no significant differences (*p >* 0.05) between each group in the α-diversity analysis.

**Figure 5:**
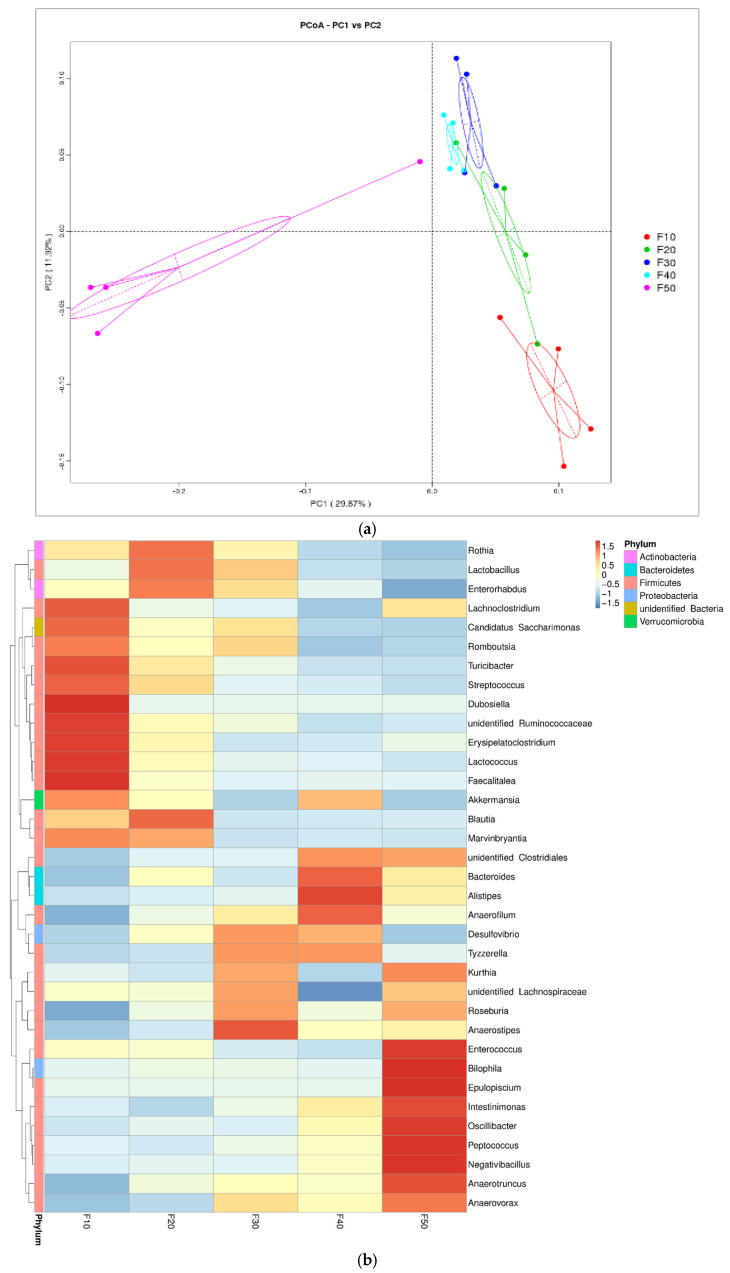
Gut bacteria composition at the genus level in SD rats fed diets with different ratios of fat/carbohydrates (10/70, 20/60, 30/50, 40/40, and 50/50) *n =* 4. (**a**) Principal coordinate analysis (PCoA) used un-weighted UniFrac distance metrics. (**b**) Hierarchical clustered heat map with Z-score normalized relative abundance of Top 35.

Principal coordinate analysis (Figure 5a) showed that the F30 (30/50) and F40 (40/40) groups had similar gut microbiota structures; however, they had dramatically changed gut microbiota patterns compared with the rats in the F10 (10/70) and F50 (50/50) groups.

At the genus level, the data of the hierarchical clustered heat map of relative abundance were normalized (Figure 5b). The horizontal direction was sample information, and the vertical direction was species annotation information. *Rothia*, *Lactobacillus*, *Enterorhabdus*, and *Blautia* were relatively abundant in group F20 (20/60), while probiotic strains such as *Lactococcus* and *Faecalitalea* were abundant in F10 (10/70). High relative abundances of *Anaerotruncus* and *Enterococcus* were found in the F50 (50/50) group.

## 4. Discussion

A systematic review illustrated that very-low-carbohydrate ketogenic diets lead to greater long-term weight loss and improved cardiovascular risk factors compared to low-fat diets, but may increase LDL-cholesterol levels [17]. And another report [18] showed that low-carbohydrate diets are associated with significant reductions in body weight and cardiovascular risk factors, although the long-term health effects remain unknown. Similarly to the above, obese patients following a very low-calorie ketogenic diet experience improvements in psychological well-being, food cravings, physical activity, and quality of life, alongside significant weight and fat mass loss [19].

According to the reports above, ketogenic diets have shown promising results in obesity treatment, body weight control, and even longevity and lifespan [20]. However, these investigations were mostly focused on individuals with some diseases, and the sample amounts remained relatively small and were studied in the short term. Moreover, as mentioned in the introduction, a strict KD was difficult to maintain for overweight populations. In this case, we modified the ratio of fat in the KD and created a “generalized ketogenic diet”.

Researchers have questioned whether the effectiveness of KD in weight control is due to the suppression of appetite from its dietary high-fat ratio. Considering that, this isoenergetic experiment was designed to exclude the influence of appetite suppression. The body weight growth of rats had no significant difference among all the groups (Figure 1). With the increasing fat/carbohydrate ratio in the diet, colon length was elongated dose-dependently. The colon length of rats in group F50 was 35% longer than that of rats in group F10 vs. F50 (Table 2), which may have induced defecation difficulties and even intestinal problems [21].

In isoenergetic circumstances, a low F/C ratio led to a high level of TG and TP (Figure 2a,b). The serum level of TG was highly related to non-communicable diseases (NCDs), which suggested that the carbohydrate dominant dietary pattern would induce an increase in TG. The serum TP level has multiple physiological functions, and it is normally used for monitoring the nutritional status of subjects. The result of TP indicated that isoenergetic KD might induce malnutrition of subjects in a long-term pattern. ALT is generally regarded as a critical indicator to evaluate hepatocyte lesion and liver function. The result showed that a high ratio of F/C diets induced liver impairment. SAP was another indicator to evaluate liver function. Inflammation of the liver may induce an SAP increase in the serum (Figure 2c,d). These results are anastomotic with the pathological section outcomes in the present study, in which the high ratio of dietary F/C caused hepatic steatosis and infiltration of inflammatory, scattered lymphocytes and narrow sinusoidal liver. Generally, in our circumstances, the isoenergetic KD (high ratio of F/C) led to liver impairment in SD rats.

In regard to gut bacteria, the relative abundance of *Actinobacteria* showed significant differences (Supplementary Figure S1) at the phylum level in the groups consuming different ratios of F/C, and had a peak in rats of the F20 (20/60) group.

All the genera that showed significant differences among groups are summarized in Table 3 (Figure 5b). Probiotics like *Lactobacillus*, *Lactococcus*, *Faecalitalea*, and *Blautia* had higher relative abundances in F10 (10/70) and F20 (20/60) than in F50 (50/50). Simultaneously, most of the harmful strains listed showed higher relative abundances in groups F10, F20, and F50 than F30 and F40. Therefore, overconsuming either a high-fat or a high-carbohydrate diet could induce the proliferation of maleficent bacteria. Inappropriate dietary patterns might lead to potential hazards in the long-term due to the disruption of gut microbiota homeostasis by accumulating maleficent bacteria.

**Figure 6:**
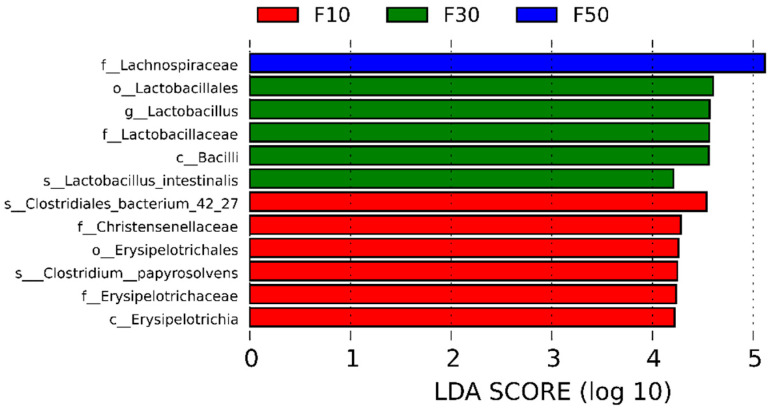
LEfSe analysis of taxonomic biomarkers of F10 (control) SD rats and SD rats fed with different ratios of fat (30% (F30) and 50% (F50)), *n =* 4.

To identify the characteristic bacteria among the groups, a linear discriminant analysis (LDA) score was applied (LDA > 4 were showed in Figure 6). *Lachnospiraceae* could be regarded as a biomarker of F50 (50/50), and an association was proven with a high risk of colon cancer, although in some circumstances, the role of *Lachnospiraceae* is not fully understood. [30]. *Erysipelotrichaceae* from F10 (10/70) was associated with inflammatory bowel diseases (IBDs) [31]. It has been reported [32] that Christensenellaceae (biomarkers of group F30) is inversely related to the host body mass index (BMI). Similarly, LDA score was in accordance with the clustered heatmap (Figure 5b), suggesting that either a low or high F/C diet altered the gut microbiota in an undesirable way. Moderating fat content helped to increase the relative abundance of probiotics in rats.

In addition, to design a set of isoenergetic diet formulas with different F/C ratios, insoluble cellulose (celluarose) was added in different amounts into each group’s feed (Table 1). And additive amounts of insoluble cellulose were increased along with the increasing ratio of fat. Insoluble cellulose has been generally regarded as a prebiotic which modulates the structure and composition of gut bacteria; reduces the risk of IBD, heart diseases and stroke, type 2 diabetes, and some cancers; and lowers blood sugar and the LDL-cholesterol level [33]. However, from our results, the gut composition of group F50 (50/50) was not beneficial; on the contrary, some inflammation-induced strains showed higher relative abundance, even though group F50 (50/50) contained the highest level of insoluble cellulose (Table 1). This indicated that the beneficial effects of dietary fiber could not be exerted while the high ratio of F/C was consumed. However, the mechanisms of the apparent results remain unclear. Body composition changes, liver inflammation biomarkers, and their regulating pathways need to be analyzed in further studies. Rational studies are useful as a scientific basis for formulating balanced diets in humans.

Based on the experimental period (8 weeks) and experimental animals (rats), our results have their own limitations. We have used healthy rats as experimental objects, but we might need to build more animal models, such as anxiety, depression, and insomnia models, to observe the generalized ketogenic diet’s risks before launching clinical trials or cohort studies.

## 5. Conclusions

The present study provides significant insights into the physiological implications of a generalized ketogenic diet for Sprague–Dawley rats. Our findings demonstrate that a diet high in fat content, specifically the 50% fat diet, can lead to mild liver damage, as evidenced by increased serum levels of alanine aminotransferase (ALT) and serum alkaline phosphatase (SAP), along with histological observations of hepatic steatosis and inflammation. Furthermore, the generalized ketogenic diet disrupted the gut microbiota equilibrium, reducing the abundance of beneficial probiotics such as *Lactobacillus*, *Lactococcus*, and *Faecalitalea* while increasing the levels of potentially pathogenic bacteria like *Anaerotruncus*, *Enterococcus*, *Rothia*, and *Enterorhabdus*. These alterations in gut microbiota could have sustaining influences, given the established link between gut health and overall well-being.

In summary, while the generalized ketogenic diet may offer short-term benefits in weight management and blood glucose control, our results suggest that adherence to such a diet could pose health risks. The observed impairments in liver function and the shift in gut microbiota composition underscore the need for caution when recommending ketogenic diets for extended periods. Future research should focus on understanding the mechanisms underlying these effects and exploring strategies to mitigate the adverse outcomes associated with high-fat diets. Until then, it is prudent to approach the generalized ketogenic diet with skepticism, particularly for those considering its use as a long-term dietary intervention.

## Figures and Tables

**Table 1:** Diet formulas and nutrients with different ratios of fat/carbohydrates (10/70, 20/60, 30/50, 40/40, and 50/50).

Items	Groups				
	Control (F10)	F20	F30	F40	F50
Energy (%)					
Ratio of fat/carbohydrates	10/70	20/60	30/50	40/40	50/50
Carbohydrates	71.00	63.00	54.00	45.00	37.00
Protein	19.00	17.00	16.00	15.00	14.00
Fat	10.00	20.00	30.00	40.00	49.00
Metabolizable energy kcal/g	3.60	3.60	3.60	3.60	3.60
Total energy kcal/g	3.87	4.12	4.42	4.79	5.23
Nutrients (%)					
Brown rice	50.00	50.70	46.33	30.92	5.00
Casein	14.13	14.05	14.56	16.36	19.39
Soybean oil	2.94	7.59	12.85	19.44	27.20
Sucrose	14.43	5.00	2.00	0.00	0.00
Dextrin	12.53	10.00	3.00	0.00	0.00
Cellulose	0.91	7.60	16.21	28.22	43.35
Vitamin premix	1.00	1.00	1.00	1.00	1.00
Mineral premix	3.50	3.50	3.50	3.50	3.50
Cysteine	0.30	0.30	0.30	0.30	0.30
Choline chloride	0.25	0.25	0.25	0.25	0.25
TBHQ	0.01	0.01	0.01	0.01	0.01
In total	100.00	100.00	100.00	100.00	100.00

**Table 2:** Tissue weight and colon length of SD rats (*n =* 10) fed diets with different ratios of fat/carbohydrates (10/70, 20/60, 30/50, 40/40, and 50/50) for 8 weeks.

Items	Groups					*p* Value
	Control (F10)	F20	F30	F40	F50	
Liver weight (g)	18.32 ± 0.86	17.45 ± 0.78	18.03 ± 0.58	18.55 ± 0.64	17.16 ± 0.81	0.938
Kidney weight (g)	3.90 ± 0.11	3.90 ± 0.13	3.81 ± 0.10	3.93 ± 0.10	4.05 ± 0.11	0.748
Epididymis fat mass (g)	11.07 ± 0.84	14.16 ± 0.90	14.19 ± 0.72	14.24 ± 0.78	13.80 ± 0.88	0.824
Relative weight tissue ^a^						
Liver	3.25 ± 0.08	2.97 ± 0.09	3.07 ± 0.09	3.11 ± 0.07	3.03 ± 0.09	0.441
Kidney	0.70 ± 0.02	0.67 ± 0.02	0.65 ± 0.01	0.66 ± 0.01	0.72 ± 0.02	0.116
Epididymis fat mass	1.96 ± 0.11	2.40 ± 0.10	2.41 ± 0.12	2.38 ± 0.10	2.44 ± 0.13	0.951
Colon length (cm)	15.41 ± 0.45 a	15.59 ± 0.78 a	18.38 ± 0.64 b	18.88 ± 0.51 b	20.85 ± 0.39 c	<0.001

Values are shown as means ± SEM, *n =* 10. Means in a row without a common letter differ, *p* < 0.05. ^a^ Tissue weight/body weight × 100.

**Table 3:** Physiological function summary of gut bacteria at the genus level.

Classification	Bacteria	Highest Group of Relative Abundance	Physiological Function	Reference
harmful	*Rothia*	F20	Induce intestinal inflammation	[22]
harmful	*Enterorhabdus*	F20	Related to obesity	[23]
harmful	*Dubosiella*	F10	Induce intestinal inflammation	[24]
harmful	*Anaerotruncus*	F50	Induce intestinal inflammation	[25]
harmful	*Enterrococcus*	F50	Induce gastrointestinal tract infection	[26]
beneficial	*Lactobacillus*	F20	Control blood glucose level	[27]
beneficial	*Lactococcus*	F10	Anti-inflammation	[25]
beneficial	*Faecalitalea*	F10	Promote insulin secretion and response	[28]
beneficial	*Blautia*	F20	Related to immune function	[29]

## Data Availability

The original contributions presented in the study are included in the article/Supplementary Materials, further inquiries can be directed to the corresponding author.

## Supplementary Materials

The following supporting information can be downloaded at: https://www.mdpi.com/article/10.3390/biology13110899/s1, Supplementary Figure S1: Relative abundance in phylum level of SD rat fed diets with different ratio of fat/carbohydrate (10/70, 20/60, 30/50, 40/40 and 50/50), *n =* 4.
