# Supplementary material for: Generalized Ketogenic Diet Induced Liver Impairment and Reduced Probiotics Abundance of Gut Microbiota in Rat
Source: Biology (Basel). 2024 Nov 4;13(11):899. doi: 10.3390/biology13110899 (PMC11591927; doi:10.3390/biology13110899)
Supplement: Supplementary file 1 [file biology-13-00899-s001.zip › biology-3256108-supplementary.pdf]

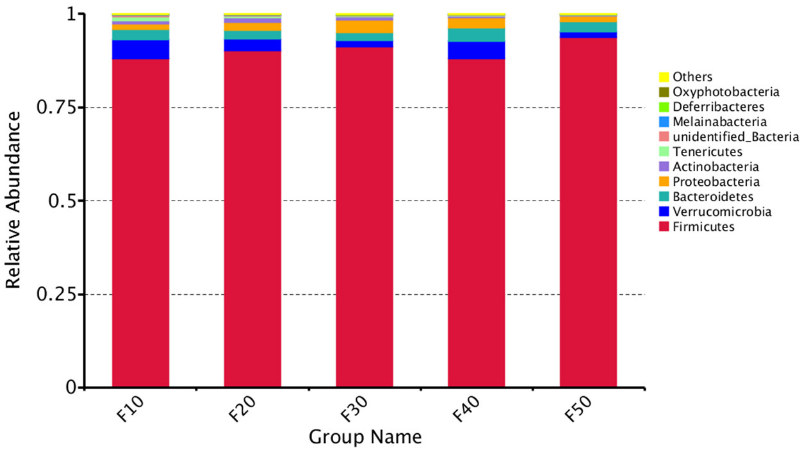

Supplementary Figure S1 Relative abundance in phylum level of SD rat fed diets with different ratio of fat/carbohydrate (10/70, 20/60, 30/50, 40/40 and 50/50), n=4
